# Effects of COVID-19-related psychological distress and anxiety on quality of sleep and life in healthcare workers in Iran and three European countries

**DOI:** 10.3389/fpubh.2022.997626

**Published:** 2022-11-25

**Authors:** Morteza Zangeneh Soroush, Parisa Tahvilian, Sepideh Koohestani, Keivan Maghooli, Nader Jafarnia Dabanloo, Mojtaba Sarhangi Kadijani, Sepehr Jahantigh, Masoud Zangeneh Soroush, Amitis Saliani

**Affiliations:** ^1^Occupational Sleep Research Center, Baharloo Hospital, Tehran University of Medical Sciences, Tehran, Iran; ^2^Bio-Intelligence Research Unit, Electrical Engineering Department, Sharif University of Technology, Tehran, Iran; ^3^Department of Biomedical Engineering, Science and Research Branch, Islamic Azad University, Tehran, Iran; ^4^Engineering Research Center in Medicine and Biology, Science and Research Branch, Islamic Azad University, Tehran, Iran; ^5^Department of Electrical Engineering, Islamic Azad University, Qazvin Branch, Qazvin, Iran; ^6^Curriculum Planning Department, Islamic Azad University, Islamshahr Branch, Islamshahr, Iran; ^7^Department Chemical Engineering, Sahand University of Technology, Tabriz, Iran; ^8^Faculty of Medicine, Shahid Beheshti University of Medical Sciences, Tehran, Iran; ^9^Department of Genomic Medicine, Queen Mary University of London, London, United Kingdom

**Keywords:** COVID-19 pandemic, sleep quality, healthcare workers, stress, anxiety, quality of life

## Abstract

**Introduction:**

The COVID-19 pandemic has considerably affected human beings most of whom are healthcare workers (HCWs) combating the disease in the front line.

**Methods:**

This cross-sectional study aims to explore the effects of stress and anxiety caused by COVID-19 on the quality of sleep and life in HCWs, including physicians, nurses, and other healthcare staff. In this global study, we asked 1,210 HCWs (620 and 590 volunteers from Iran and European countries, including Germany, the Netherlands, and Italy, respectively), who age 21–70, to participate in the test. Several measures of COVID-related stress, anxiety, sleep, and life quality, including the 12-item General Health Questionnaire (GHQ-12), Fear of COVID-19 scale (FCV-19S), Beck Anxiety Inventory (BAI), the Pittsburgh Sleep Quality Index (PSQI), and World Health Organization Quality of Life-BREF (WHOQOL-BREF) are recorded.

**Results:**

Volunteers reported high rates of stress and anxiety and poor sleep quality as well as lower quality of life. The correlation analysis between the measures is reported. According to the results, regardless of the location, HCWs, predominantly female nurses, developed anxiety and stress symptoms which consequently resulted in lower sleep and life quality. Both for Iranian and the European HCWs, significant differences existed between nurses and the other two groups, with the *p*-values equal to 0.0357 and 0.0429 for GHQ-12, 0.0368, and 0.714 for BAI measure. Even though nurses reported the most stress, anxiety, fear of COVID-19, lower quality of life and sleep in both countries, and also an increase in other measures as well, there existed no statistically significant difference in FCV-19S, PSQI, and WHOQOL-BREF.

**Discussion:**

This study helps to expand our knowledge the effects of pandemics on HCWs and also for healthcare management to predict HCW's mental health conditions in similar situations.

## Introduction

A large virus family named Coronaviruses, which can cause different conditions such as the common cold to more severe forms of diseases like Middle East Respiratory Syndrome (MERS-CoV) and Severe Acute Respiratory Syndrome (SARS-CoV), have been at the center of attention for more than 30 months (in 2020, 2021, and 2022) (1–5). A worldwide pandemic has been developed by a novel coronavirus coming from Wuhan, China. Severe Acute Respiratory Syndrome-Coronavirus-2 (SARS-CoV-2) has led to an unbelievable challenge for the health community all over the world. The first cases were detected in December 2019. Then, due to the virus's asymptomatic transmission ability, it reached almost all countries all over the world (6–10). The COVID-19 pandemic, with over 605 million cases and 6 million deaths worldwide, is a global health issue now. At the time of writing, more than 605,718,064 COVID-19 cases and 6,487,773 deaths have been reported, while the real numbers are way larger. Almost the same numbers are reported by www.worldometers.info, The New York Times (www.nytimes.com), JHU CSSE COVID-19 Data (https://github.com/CSSEGISandData/COVID-19), and Our World in Data (www.ourworldindata.org) (11–18).

Governments have taken different approaches, such as spatial distancing, quarantining, and lock-down, which have totally affected human beings (19–23). Several countries have imposed a national lockdown as the main course of action for some periods. The precautions and precautionary behaviors have interfered with the lives of people resulting in the health and prosperity of individuals (24–29). These policies, which have considerably changed our daily life, have resulted in stress and anxiety among communities. Several studies have reported that COVID-19 and the mandatory lockdown cause anxiety, stress, and sleep quality. As there is still no specific treatment, countries are forced to follow strict obligations and prohibitions such as isolation which may keep individuals physically well but they are psychologically challenged (19–37).

Healthcare workers (HCWs), as part of society, are no exception. Due to limited knowledge about the disease, unknown surefire treatment for it, the workload in healthcare centers and referral hospitals, etc. HCWs have been under considerable mental pressure resulting in psychological distress, anxiety, and poor sleep quality (1, 2, 8, 19, 20, 32, 33). Surveys have claimed that in most countries, healthcare professionals are under overwhelming psychological stress. Referring to psychologists and psychiatrists has considerably increased (20, 23, 27, 32, 33). Caregivers are exposed to mental pressure as they witness death every single day for a long time (32, 33). HCWs are also exposed to unpredictable events in their workplace due to this ongoing and challenging crisis. They are somehow deprived of their basic needs, for instance, meeting their family members to keep them safe and not to transmit the virus (1, 20, 22, 32–35). This again worsens the situation for HCWs. They face an extraordinary workload in their workplace. In most cases, they get infected and need to stay at home for some days, which imposes even more mental and physical pressure on their colleagues. It is reported that the recent pandemic has led to stress, anxiety, depression, insomnia, anger, poor sleep quality, and fear among HCWs (1, 2, 21, 34, 35). For healthcare professionals, stress and anxiety are the most critical factors which have seriously aggravated the quality of life and sleep. These factors consequently affect their work performance and mental ability (32–37). Moreover, HCWs have worked in adverse working conditions which have considerably affected their mental health and most likely affected their wellbeing for a long time. Several studies such as (38–44) have reported that working condition is the most predominant moderator of anxiety, fear, quality of life, and sleep in HCWs during the COVID-19 pandemic. Due to the adverse working conditions and their high workload, HCWs formed the highest levels of anxiety and fear compared to other groups in society. There is a high presence of symptomatology related to work stress for HCWs every day, which consequently has resulted in physical and emotional fatigue, overload, tension, and anxiety. Several studies have reported impaired mental health among HCWs during the pandemic (20, 34, 45–52). For example, in England, there was an increased prevalence of mental health disorders potentially sufficient to impair high-quality care delivery (45). Nurses working in intensive care units (ICU) were functionally impaired by the state of their mental health and reported higher rates of symptoms consistent with common mental disorders and post-traumatic stress disorder (PTSD) compared with other ICU staff. About 25% of HCWs have reported clinically elevated anxiety and depression during the COVID-19 pandemic (46–49). Several symptoms related to impaired mental health have been reported, including probable major depression, anxiety, posttraumatic stress disorder (PTSD), alcohol-use problems, lower team cohesion, and difficulty following hospital policies (20, 34, 50–52). To the best of our knowledge, a few studies such as (1, 2) have specifically explored the differences in mental pressure and workload between physicians, nurses, and other health care workers comprehensively. They reported that sleep disturbances and low quality of life were more prevalent among nurses compared to the other two groups. This is inconsistent with our findings in the present study. However, more exploration is required to completely study the differences between the aforementioned three groups of HCWs. This motivated us to conduct this study to compare these three groups considering several measures.

Strong associations between gender and COVID-19-related stress, fear, and anxiety have been reported by several studies. Generally, females consider this pandemic a more serious threat to personal health and the population compared to males (53–65). In some studies, such as Metin et al. (53), a meta-analysis was conducted to explore gender differences in terms of stress, anxiety, and fear caused by the COVID-19 pandemic. It was reported that statistically speaking, gender has a moderate effect on COVID-19 anxiety and fear in females. The findings in Metin et al. (53), as the first and most extensive meta-analysis in terms of gender differences in COVID-19-related anxiety and fear, are in agreement with ours in the present paper. Authors in Metin et al. (53) reviewed the effects of gender in different groups, including ordinary ones and HCWs. It was reported that the continent variable was a statistically significant moderator of gender difference which suggests that the location should be considered an important factor. Previous studies have disclosed inconsistent findings about fear and anxiety caused by the COVID-19 pandemic across different populations and regarding gender differences. On the other hand, some studies such as (53–65) reported that males had higher anxiety and fear levels in comparison to females. Surprisingly, some studies like (64, 65) reported no significant difference in terms of COVID-19 anxiety and fear between males and females. This motivated us to consider gender in our present study as well as other factors to clarify gender differences in this matter. Moreover, we have tried to compare several factors between Iranian and European HCWs to have a more comprehensive view of this study. It is worth mentioning that the abovementioned studies have targeted different groups in society rather than HCWs. To the best of our knowledge, the present paper is the first and the most extensive study exploring several parameters and moderators such as gender, nationality, sociodemographic information, etc. in terms of anxiety, stress, fear caused by the pandemic as well as the quality of life and sleep among HCWs.

The lockdown measures, workload, anxiety, and stress caused by this pandemic are highly associated and consequently result in sleep disturbance (26, 36, 37, 66). Wellbeing is affected by psychological problems. Experts believe that stress and anxiety can lead to severe mental and physical disorders and diseases over long periods of time. Long-term stress can lead to serious diseases and disorders such as different types of cancer. As it was mentioned above, the pandemic has affected human beings, specially HCWs (66, 67). Similar to human experience in previous pandemics, for HCWs, stress, anxiety, workload, etc. have caused intense emotional adaptation reactions, depressive symptoms, anger, various degrees of anxiety disorders, guilt, posttraumatic stress disorders, perception of grief and loss, different levels of psychological problems such as aggression, stigmatization, attention deficit, and sleep problems (1, 2, 26, 27). Some studies have tried to assess mental health in the pandemic in different regions all over the world and have concluded that COVID-19, the mandatory lockdown, and people's exposure (including HCWs) to the sudden onset of an unknown disease with a high mortality rate have affected anxiety, stress, and sleep quality (1, 2, 8, 26, 27, 36, 37).

Sleep is a biological imperative playing a crucial role in health and wellbeing. Stress and sleep quality are two important factors in one's quality of life. Previous studies suggest that sleep, stress, and anxiety are reciprocally connected (8–15). Higher waking cortisol is associated with lower sleep quality. There are also comprehensive studies showing that psychological stress modulates several of the same immunologic pathways which are observed in sleep research. The level of stress, directly, and indirectly, affects health in several ways such as weakening the immune system, poor sleep quality, short sleep duration, insomnia, etc. (11–14). Different factors including energy expenditure, substances consumed, and psychological stress determine one's sleep quality. Although a few studies have claimed that the pandemic's effects on sleep are inconclusive, several previous ones have reported that individuals had poor sleep quality and were found to be going to bed and waking up later than usual, and it again plays a crucial role in human's immune system, performance, mood, and anxiety (1–8). Sleep quality plays an important role in mental health. Poor sleep quality and irregular sleep patterns are associated with trauma and significant stressors. In most recent studies, HCWs have claimed poor sleep quality during the pandemic. Sleep quality has a direct effect on the quality of life which is vital for healthcare workers, especially in critical situations like pandemics (19, 20, 33, 34, 36). Quality of sleep and consequently quality of life affect the quality of patient care, tolerance, effective function, precision, and job satisfaction. Quality of life has dramatic effects on how an individual thinks, behaves, feels, and solves problems. It can also affect one's performance and might lead to losing their job, which again aggravates one's health condition and mental health (1–5). Needless to say, stress and anxiety, which are two major effects of the recent pandemic, are linked to the quality of sleep and consequently quality of life. Taking a closer look, it seems that there is a circular causality between the side effects of the pandemic, one's psychological stress, anxiety, one's quality of sleep, and their quality of life (2, 8, 16, 23, 28, 29, 35, 36).

All aforementioned reasons suggest that studying the effects of stress and anxiety, caused by the COVID-19 pandemic in HCWs, on their quality of sleep and life is of high importance. This motivated us to conduct this study. Considering the current situation in most countries and also the importance of this issue in health systems management, only a few studies have been conducted so far. It means that we need to view this topic from different aspects. Some studies have targeted the same issue during the pandemic. Authors in Korkmaz et al. (1), recorded data from 140 HCWs, in Turkey, including their sociodemographic data, Pittsburgh sleep quality index (PSQI), Problem-solving inventory (PSI), World health organization quality of life-BREF (WHOQOL-BREF)—short version, and Beck anxiety inventory (BAI). There was no significant difference in BAI for all three groups. Nurses had higher PSQI and PSI scores. The quality of life scores of the nurse participants was also lower. In a similar study conducted in Nigeria, information from 303 HCWs was recorded and analyzed. The data included Sociodemographic data, the 12-item general health questionnaire (GHQ-12), and the Pittsburgh Sleep Quality Index (PSQI). About 23.4% of the HCWs reported psychological distress, 60% reported sleep problems, and psychological distress correlated significantly with poor sleep. In China (8), 323 HCWs were studied, and data included The 25-item Chinese version nurses' occupational stressor scale (NOSS), the 7-item Cognitive Fusion Questionnaire (CFQ), the 6-item cognitive reappraisal subscale of the Emotion Regulation Questionnaire (ERQ), the Chinese version of the 12-item General Health Questionnaire (GHQ-12), the 18-item Pittsburgh Sleep Quality Index (P, SQI), and demographic variables. Nurses' occupational stressors directly linked to mental health problems, Occupational stressors significantly linked to nurses' mental health problems, and cognitive fusion and cognitive reappraisal of nurses significantly mediated the links from occupational stressors to mental health problems. These recent studies explored the effects of stress and anxiety caused by the COVID-19 pandemic on the quality of sleep and life.

As it can be seen, in these studies only one or two countries have been considered and fewer than 400 HCWs have participated. To have a wider view, it is necessary to conduct a more comprehensive study including more countries and a larger sample population. In some recent studies, only a limited number of a specific group of HCWs (like nurses) are just considered. As we know, all HCWs in the whole healthcare system are involved more or less and are under mental and physical pressure. So, we need to consider a larger sample population, including all groups of healthcare givers. In some studies, only one measure is used to assess the quality of life, the level of anxiety, etc. It is vital to utilize different assessment methods and factors to evaluate the effects of stress and anxiety fairly. So, the abovementioned issues motivated us to conduct the present study. We have employed different measures to analyze the problem among HCWs, including nurses, physicians, and other healthcare staff. Moreover, we spent almost 2 months collecting data in 2020 to have a more comprehensive study. Considering all the aspects, this study has made a significant contribution to future studies and our understanding of the recent pandemic and its effects on an extensive community like health care workers.

In this paper, we are going to study the level of stress and anxiety among HCWs and the correlation between these factors and the quality of life and sleep. In the present study, we explore the association between stress caused by the COVID-19 pandemic and the quality of sleep and life in healthcare workers in Iran and the above-mentioned European countries. Our main aim is to study and compare the effects of stress and anxiety caused by the pandemic on the quality of sleep and life in the aforementioned groups among a large, international community sample.

## Materials and methods

### Participants

We collected data in 2 months, from September 2020 to October 2020 (during the severest period of the COVID-19 pandemic). This cross-sectional and descriptive study was conducted with the approval of the local ethics committee. The approval was obtained from the local ethics committee and in agreement with the Helsinki Declaration. All volunteers were asked to study our project's objectives and to read and sign a written consent on our forms before answering the questions. They were informed about the study and its goals and then were asked to read and sign a written consent. All forms, questionnaires, documents, and questions were translated into English and Farsi by the same translator who is also an expert in psychology. Our study inclusion criteria were signed informed consent, having been working as an HCW to combat COVID-19 for at least 2 months during the pandemic, aged between 20 and 70, and having Farsi or English literacy. All participants were asked to choose either Farsi or English to complete the forms. Some participants were excluded from the study due to their limited knowledge of Farsi or English languages. The included participants declared intermediate or advanced knowledge (native speakers) in English or Farsi. With the aim of decreasing the spread of COVID-19 disease and also to have data from two countries, we decided to collect data using Google forms on online platforms and social media.

We employed the method of sample calculation for an unknown population and the following formula to calculate the sample size in our study (5, 19, 37).


(1) $$\begin{array}{l} n=t^{2*}p^{*}q^{*}d^{2} \end{array}$$

where *t* is the value on the t-table at a certain degree of freedom and predetermined error rate. p and q represent the prevalence of the event and the prevalence of the absence of the event, respectively. *d*^2^ shows the deviation to be achieved based on the prevalence of the event. Our estimate was 593 for the number of individuals to be included. We used a confidence interval of 0.95%, 50% unknown prevalence, and a standard deviation of 5%.

Almost 2,000 HCWs from Iran and European countries participated in our experiment as we tried our best to share the link of our Google forms in the best possible way. We employed social media (Facebook, Telegram, Instagram, and WhatsApp) to share study information and links with volunteers. We asked our colleagues working in foreign countries to help us and share the links as much as they could. Due to the number of participants and missing information in some cases, 1,210 forms were approved.

The total numbers of participants were 799 and 723 HCWs in Iran and the studied European countries. It should be noted that the total numbers of approved cases were 620 for Iran and 590 for the aforementioned European HCWs. The response rates were 77.6 and 73.8% for Iran and the before-mentioned European countries, respectively. As the main reasons, in most failed cases, HCWs claimed that they had a problem with their internet connection while accessing the forms, or they did not meet the study criteria, or they were not able to fill in the forms on their cellphones, or they were not satisfied with the number of questions. In addition, we considered mental or physical disorders and records of psychiatric treatment as the participant exclusion criteria.

Our protocol mainly includes preparing questionnaires in both English and Farsi, targeting potential volunteers in social media and networking applications, sending requests to a population of HCWs in the target countries, waiting for their responses and providing more information if required, collecting and preprocessing data, removing missing data and outliers, preparing the dataset for main analyses, conducting analysis, and reporting the results. These were the main steps that we took in this study.

Table 1 represents the distribution of the participants with respect to their gender and age in Iran and the European countries in this study.

**Table 1:** The number of male and female HCWs, in each age group, who participated in our study in Iran and the European countries.

**Samples**	**21–30**	**31–40**	**41–50**	**51–60**	**61–70**	**Total**
Male HCWs, Iran	66	99	70	67	10	312
Female HCWs, Iran	55	79	96	40	38	308
Male HCWs, European Countries	81	68	65	52	25	291
Female HCWs, European Countries	31	93	89	70	16	299
Male HCWs, Iran and European Countries	147	167	135	119	35	603
Female HCWs, Iran and European Countries	86	172	185	110	54	607
All HCWs participated in this study	233	339	320	229	89	1,210

We selected 1,210 healthcare workers, out of 2,000, who worked in the departments or clinics combating COVID-19 and who met the study criteria. Participants provide health services for COVID-19 patients in different departments and have direct contact with COVID-19 patients. We tried to have almost the same sample size in both populations in order to compare the results fairly. From Iran, 620 HCWs including 179 physicians (Phs), 302 nurses (Nus), and 139 other healthcare staff (OHS) were considered for analysis. About 590 European HCWs including 185 physicians, 278 nurses and 127 other healthcare staff were considered. Both Iranian and European HCWs were working in COVID-19 departments, clinics, and wards. They had a close contact with patients. In both societies, the numbers of male participants outweigh the number of female participants. In Iran and in the European countries, 312 male HCWs (~50.3%) and 301 male HCWs (~51%) were selected in this study, respectively. For Iranian HCWs, the average age was 38.66 ± 9.1 years and in more detail for Iranian physicians, nurses, and OHS, it was 41.3 ± 7.8, 36.9 ± 8.2, and 37.8 ± 7.6, respectively. The average age among European HCWs who participated in this study was 38.8 ± 7.9 years. The average age for European physicians, nurses, and OHS was 40.1 ± 8.3, 38.4 ± 7.9, and 37.9 ± 6.4, respectively. Volunteer HCWs were literate and between the ages of 20 and 70. All participants were asked to submit their sociodemographic and clinical information and also to fill in the forms including the Fear of COVID-19 scale (FCV-19S), Pittsburgh Sleep Quality Index (PSQI), 12-item General Health Questionnaire (GHQ-12), World Health Organization Quality of Life-BREF (WHOQOL-BREF)—Short Version, Beck Anxiety Inventory (BAI). We employed SPSS Version 22 to perform statistical analysis.

### Ethical considerations

Institutional Review Board (IRB) approval was obtained before the study commenced. Research Ethics Committee of Islamic Azad University—Science and Research Branch found our study to be in accordance with ethical principles. The approval ID is IR.IAU.SRB.REC.1400.260. For participants' identities to be confidentially safe, we associated participants with some random pre-generated codes. These codes replaced the names and identities of participants in all the further steps and analyses. The recorded data are kept in a confidential and safe repository.

### Measures

#### Sociodemographic and clinical data

We employed Google forms which had been previously prepared by the authors and are acknowledged in the literature. Our forms include socio-demographic and clinical information like age, sex, educational level, marital status, chronic illness, type of household, children, work shifts, type of workplace, their experience, their family, a record of COVID-19 in their family, death caused by COVID-19 in their immediate family, etc.

#### Fear of COVID-19 scale (FCV-19S)

In (6, 66), FCV-19S is introduced and used as a seven-item measure to evaluate the level of COVID-19 fear. This measure has good reliability, construct, and concurrent validity. FCV-19s includes seven items with a five-point Likert scale from 1 (totally disagree) to 5 (totally agree) for each item. The range of scores is 7–35. Higher scores show greater COVID-19 fear. We employed the translated version of this measure with permission from the corresponding authors to assess the level of COVID-19 fear among Iranian HCWs. FCV-19S had great internal consistency reliabilities in our study (α = 0.893, ω = 0.896).

#### Pittsburgh sleep quality index (PSQI)

The Pittsburgh Sleep Quality Index (PSQI) is a 24-item standardized self-report assessment tool with seven subscales which is used in both clinical and non-clinical applications. PSQI is employed to diagnose sleep problems and to measure sleep difficulties over a 1-month interval. The test includes 24 questions, 19 questions of which are self-report and assess subjective sleep quality. Five questions are answered by a partner or roommate. We used these 24 questions in a four-point scale (0–3) to measure sleep quality in this study. These questions give scores for sleep quality, sleep latency, sleep duration, habitual sleep efficiency, sleep disturbance, use of sleeping medication, and daytime dysfunction. Participants should answer questions through a scoring scale between 0 and 3. All scores in all subdimensions are summed up resulting in the overall index score equal to 21. Scores >5 show low sleep quality or a disturbance in sleep quality. The higher the overall index, the poorer the sleep quality. PSQI is a reliable international scale that is widely used to measure subjective sleep quality (1, 2, 4–8). The internal consistency of PSQI was 0.83 (Iran) and 0.85 (European countries). In PSQI, the diagnostic sensitivity and the specificities are 89.6 and 86.5%, respectively (67, 68).

#### 12-item general health questionnaire (GHQ-12)

GHQ-12 is a 12-item subjective questionnaire that is employed to measure psychological distress or general wellbeing (69). A bimodal scoring scale (0-0-1-1) is used and the total score ranges from 0 to 12. GHQ-12 includes six positive and six negative subscales. For positive items, scores range from 0 (always) to 3 (never), while for negative items, scores are between 0 (never) and 3 (always). Scores above 2 are assumed as indicatives of a decrease in mental health. A higher score shows severer psychological distress (69–72). GHQ-12 has been widely used almost all over the world (2, 8). In this study, the internal consistency reliability (Cronbach's α) was 0.87% for Iran and 0.86% for the above-mentioned European countries.

#### World Health Organization quality of life-BREF (WHOQOL-BREF)—Short version

World Health Organization (WHO) defines health as “A state of complete physical, mental, and social wellbeing not merely the absence of disease ....”. WHOQOL-BREF is a self-report assessment tool that is cross-culturally applicable and has been introduced by WHO in order to measure the quality of life (1, 73, 74). In the present study, participants were asked to answer the questions considering their last 15 days. In this questionnaire, there are 26 tests including two general questions and 24 specific questions targeting physical health (seven tests), mental health (six tests), social relations (three tests), and environmental health (eight tests). The scores for each subdimension range from 4 to 20, 4 indicates the lowest quality of life while 20 suggests the best quality of life. Higher scores are indicative of higher levels of quality of life.

#### Beck anxiety inventory (BAI)

Beck Anxiety Inventory (BAI) is a subjective three-point measure that is used to evaluate the frequency of anxiety which is experienced in daily life by an individual. BAI was first introduced by Beck et al. (75). Each item on this questionnaire ranges from 0 (not at all) to 3 (always) resulting in 63 scores for the whole test. Scores in the ranges of 0–7, 8–15, 16–25, and 26–63 suggest minimal, mild, moderate, and severe levels of anxiety in participants. Higher scores are indicative of higher levels of anxiety. Several studies like (1, 76, 77) have employed this measure in both Iranian and European societies and have claimed high sensitivity, specificity, and internal consistency reliability. Numerous studies have suggested BAI as an accurate and reliable measure of anxiety symptoms in children and adults (78). Internal consistency for the BAI (Cronbach's α) was 91%.

### Statistical analysis

We employed Statistical Package for Social Sciences (SPSS 26) for statistical analysis. Arithmetic means, standard deviation, percentage, and frequency were calculated using SPSS 22 to analyze our data. A one-way analysis of variance (One-Way ANOVA) and *t*-test was performed for independent samples for more than two and two independent groups, respectively. Significant differences were determined with respect to the significance interval (*p* < 0.05). In addition, we utilize Pearson correlation analysis to test the correlation between the measures. To test the normality of recorded scores we used the Kolmogorov–Smirnov test.

## Results

**Figure 1:**
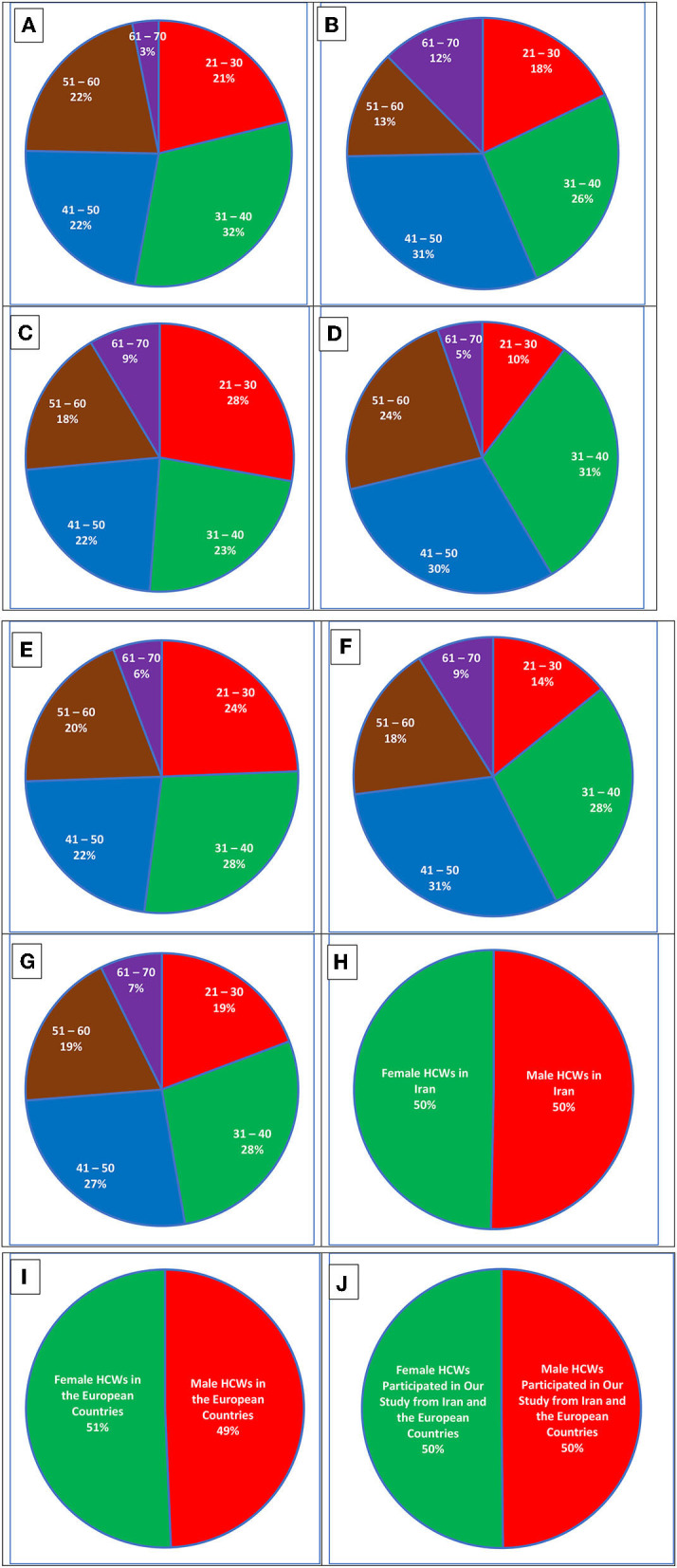
Pie charts representing more details about the participants. **(A)** Male HCWs from Iran in our previously considered age groups. **(B)** Female HCWs from Iran in age groups. **(C)** Male HCWs from the studied European countries. **(D)** Female HCWs from the European countries. **(E)** The total number of male HCWs in Iran and the European countries. **(F)** The total number of female HCWs in Iran and the European countries. **(G)** Sum of all HCWs who participated in this study from Iran and the European countries. **(H)** The number of male participants vs. the number of female participants in Iran. **(I)** The number of male participants vs. the number of female participants in the European countries. **(J)** The total number of all male participants vs. the total number of all female participants in Iran and the European countries.

This study aims to explore anxiety, psychological stress, quality of life and sleep among HCWs through sociodemographic and work-related characteristics which are represented in Table 2. During the period of our study, about 22 cases among 1,210 HCWs were identified with evidence of COVID-19 and a prevalence of 1.81% (95% CI, from 1.45 to 2.53). We also identified 73/1,210 (6.03, 95% CI, from 5.41 to 6.73). HCWs with incident COVID-19 during our data-gathering phases. The number of incident cases increased with the rising prevalence of COVID-19. Figure 1 illustrates more details about the participants and sample size in each age group using pie charts. It gives more details about male and female participants with respect to the age groups and place of living.

**Table 2:** Sociodemographic, medical, and work-related characteristics of the participants in the present study.

		**Iran**				**The studied European Countries**			
**No**.	**Characteristics**	***n***	**Phs**	**Nus**	**OHS**	***n***	**Phs**	**Nus**	**OHS**
1	**Number of participants**	620	179 (28.9%)	302 (48.7%)	139 (22.4%)	590	185 (31.4%)	278 (47.1%)	127 (21.5%)
1	**Age**		41.3 ± 7.8	36.9 ± 8.2	37.8 ± 7.6		40.1 ± 8.3	38.4 ± 7.9	37.9 ± 6.4
2	**Gender**	
	Male	312 (50.3%)	83 (46.3%)	149 (49.3%)	80 (57.5%)	301 (51.0%)	87 (47.0%)	149 (53.6%)	65 (51.1%)
	Female	308 (49.7%)	96 (53.6%)	153 (50.6%)	59 (42.4%)	289 (49.0%)	98 (53.0%)	129 (46.4%)	62 (48.8%)
3	**Educational level**	
	Primary school and high school	12 (1.9%)	0 (0%)	0 (0%)	12 (8.6%)	51 (8.7%)	0 (0%)	20 (7.2%)	31 (24.4%)
	University	608 (98.1%)	179 (100%)	302 (100%)	127 (91.4%)	539 (91.3%)	185 (100%)	258 (92.8%)	96 (75.6%)
4	**Marital status**	
	Single	221 (35.6%)	54 (30.1%)	119 (39.4%)	48 (34.5%)	229 (38.8%)	27 (14.6%)	173 (62.2%)	29 (22.9%)
	Married	399 (64.3%)	125 (69.9%)	183 (60.6%)	91 (65.4%)	361 (61.2%)	158 (85.4%)	105 (37.8%)	98 (77.1%)
5	**Chronic illness**	
	Yes	121 (19.5%)	23 (12.8%)	61 (20.2%)	37 (26.6%)	71 (12.0%)	13 (7.0%)	46 (16.5%)	12 (9.4%)
	No	499 (80.5%)	156 (87.2%)	241 (79.8%)	102 (73.3%)	519 (88.0%)	172 (93.0%)	232 (83.5%)	115 (90.6%)
6	**Type of household**	
	Family	402 (64.8%)	112 (62.6%)	204 (67.5%)	86 (61.8%)	364 (61.7%)	136 (73.5%)	123 (44.2%)	105 (82.6%)
	Alone	128 (20.6%)	38 (21.2%)	53 (17.5%)	37 (26.6%)	164 (27.8%)	37 (20.0%)	114 (41.0%)	13 (10.2%)
	Friends	90 (14.5%)	29 (16.2%)	45 (14.9%)	16 (11.5%)	62 (10.5%)	12 (6.4%)	41 (14.7%)	9 (0.7%)
7	**Have children at home**	
	Yes	269 (43.3%)	132 (73.7%)	92 (30.4%)	45 (32.3%)	260 (44.1%)	109 (58.9%)	84 (30.2%)	67 (52.8%)
	No	351 (56.7%)	47 (26.2%)	210 (69.5%)	94 (67.6%)	330 (55.9%)	76 (41.1%)	194 (69.8%)	60 (47.2%)
8	**Rotating work shifts**	
	Yes	387 (62.4%)	123 (68.7%)	237 (78.4%)	27 (19.4%)	319 (54.1%)	97 (52.4%)	207 (74.4%)	15 (11.8%)
	No	233 (37.6%)	56 (31.3%)	65 (21.6%)	112 (80.6%)	271 (45.9%)	88 (47.6%)	71 (25.5%)	112 (88.1%)
9	**Record of COVID-19 diagnostic**	
	Yes	510 (82.3%)	124 (69.3%)	268 (88.7%)	118 (84.9%)	418 (70.1%)	139 (75.2%)	186 (66.9%)	93 (73.2%)
	No	110 (17.7%)	55 (30.7%)	34 (11.3%)	21 (15.1%)	172 (29.1%)	46 (24.8%)	92 (33.1%)	34 (26.8%)
10	**COVID diagnosed in close family/friends**	
	Yes	564 (90.9%)	161 (89.9%)	284 (94.3%)	119 (85.6%)	521 (88.3%)	167 (90.2%)	253 (91.0%)	101 (79.5%)
	No	56 (9.1%)	18 (10.1%)	18 (5.9%)	20 (14.3%)	69 (11.7%)	18 (9.8%)	25 (9.0%)	26 (20.5%)
11	**Death (in immediate family members due to COVID)**		
	Yes	55 (8.8%)	13 (7.2%)	29 (9.6%)	13 (9.4%)	55 (9.3%)	19 (10.2%)	17 (6.1%)	19 (14.9%)
	No	565 (91.2%)	166 (92.7%)	273 (90.3%)	126 (90.6%)	535 (90.7%)	166 (89.8%)	261 (93.9%)	108 (85.1%)
12	**Alcohol use**	
	Yes	272 (43.8%)	66 (36.8%)	139 (46.0%)	67 (48.2%)	348 (58.9%)	121 (65.4%)	170 (61.2%)	57 (44.8%)
	No	348 (56.2%)	113 (63.2%)	163 (54.0%)	72 (51.8%)	232 (39.3%)	54 (29.2%)	108 (38.8%)	70 (55.1%)
13	**Tobacco use**	
	Yes	241 (38.8%)	49 (27.3%)	96 (31.8%)	96 (69.0%)	176 (29.8%)	53 (28.6%)	69 (24.8%)	54 (42.5%)
	No	379 (61.1%)	130 (72.6%)	206 (68.2%)	43 (31.0%)	414 (70.2%)	132 (71.4%)	209 (75.2%)	73 (57.4%)
14	**Having a pregnant family member at home**	
	Yes	81 (13.0%)	42 (23.4%)	13 (4.3%)	26 (18.7%)	87 (14.8%)	49 (26.4%)	31 (11.1%)	7 (5.5%)
	No	539 (87.0%)	137 (76.6%)	289 (95.7%)	113 (81.3%)	503 (85.2%)	136 (73.6%)	247 (88.9%)	120 (94.5%)
15	**Having a family member with a chronic illness at home**	
	Yes	121 (19.5%)	17 (9.4%)	86 (28.4%)	18 (12.9%)	61 (10.3%)	21 (11.3%)	29 (10.4%)	11 (8.6%)
	No	499 (80.4%)	162 (90.6%)	216 (71.5%)	121 (87.1%)	529 (89.7%)	164 (88.7%)	249 (89.5%)	116 (91.4%)
16	**Having a family member at the age of or older than 65 years at home**	
	Yes	244 (39.3%)	74 (41.3%)	134 (44.3%)	36 (25.9%)	150 (25.4%)	51 (27.5%)	63 (22.6%)	36 (28.4%)
	No	376 (60.6%)	105 (58.7%)	168 (55.7%)	103 (74.1%)	440 (74.6%)	134 (72.5%)	215 (77.4%)	91 (71.6%)
17	**Year of experience in profession**	
	<1 year	99 (15.9%)	39 (21.8%)	46 (15.2%)	14 (10.0%)	61 (10.3%)	11 (5.9%)	29 (10.4%)	21 (16.5%)
	1–10 years	373 (60.1%)	102 (56.9%)	179 (59.2%)	92 (66.2%)	419 (71.0%)	127 (68.6%)	208 (74.8%)	84 (66.1%)
	>10 years	148 (23.8%)	38 (21.2%)	77 (25.5%)	33 (23.7%)	110 (18.6%)	47 (25.4%)	41 (14.7%)	22 (17.3%)
18	**Workplace**	
	Department	122 (19.6%)	25 (13.9%)	54 (17.8%)	43 (30.9%)	187 (31.7%)	39 (21.1%)	105 (37.7%)	43 (33.8%)
	Clinic	172 (27.7%)	48 (26.8%)	85 (28.1%)	39 (28.0%)	154 (26.1%)	47 (25.4%)	68 (24.4%)	39 (30.7%)
	Department + Clinic	326 (52.5%)	106 (59.2%)	163 (53.9%)	57 (41.0%)	249 (42.3%)	99 (53.5%)	105 (37.7%)	45 (35.4%)

As can be seen in Figure 1, in Iran, HCWs between 30 and 50 mostly participated in the experiment. While in the before-mentioned European countries, other age groups have more in number. In Iran, there are also considerable differences between male and female participants. For example, the (61–70) age group includes just 3% of male HCWs and 12% of female Iranian HCWs. This difference between male and female HCWs follows almost the opposite trend for Iranian participants in the (51–60) age group where the number of male HCWs outweighs the number of female ones. Young male HCWs in Iran, who are between 20 and 40, were more interested in participating in the experiment while mid-age and old female HCWs, who are between 40 and 70, showed more participation in our experiment. The same pattern also applies to European female HCWs. In the European countries, however, there is no considerable difference between young HCWs and mid-age ones. To better understand, the differences between male and female HCWs in the full sample and between age groups, we need to take a look at Figures 1E–G which represent the total number of male HCWs in Iran and the aforementioned European countries, the total number of female HCWs in Iran and the European countries, and all HCWs who participated in this study from Iran and the European nations, respectively.

In Iran, for male HCWs, the oldest age period (61–70) had the least participation at about 3%. However, female Iranian HCWs showed 12% of participation which is considered a significant difference. It could be due to early retirement or less interest in filling out online questionnaires by this age group. In the studied European countries, this age group again showed the least participation with 5% for male and 9% for female HCWs suggesting a considerable difference between male and female HCWs in participation. In addition, it can be seen that while in Iran and the studied European countries, younger male HCWs [in the age gaps of (21–30) and (31–40) years] participated in our study, female HCWs of older ages participated in our project.

According to data collected in our study and Table 2, in terms of tobacco use, out of 241 (38.8%) Iranian HCWs who smoke, 115, 89, and 37 participants reported an increase in daily consumption, lower consumption, and no change in their daily consumption, respectively. About 27.3% of Iranian physicians, 31.8% among Iranian nurses, and 69.0% of Iranian OHS, who participated in this study, have reported tobacco use during the pandemic. The majority of Iranian HCWs who reported an increase in tobacco daily consumption were nurses and OHS who mostly work rotatory shifts in both outpatient clinics and departments. There is no significant difference (*p* = 0.186) between HCWs who smoke in terms of the place of service. There was a statistically significant difference (*p* = 0.041) between physicians and the other two groups of Iranian HCWs with increased use of tobacco. Marital status, gender, educational level, lifestyle, having children, having a family member with chronic illness, having a family member at the age of or older than 65 years at home and professional experience did not affect the use of tobacco in Iranian HCWs. Smokers with a chronic illness or with a pregnant family member reported lower consumption during the pandemic.

In the studied European countries, among 176 smokers (29.8%), 84 participants had an increased tobacco consumption, 56 reported a decreased consumption and 36 European HCWs reported no change in daily tobacco consumption. About 19.6% of physicians, 41.8% of US nurses, and 49.0% of European OHS, who participated in this study, have reported tobacco use in the pandemic. Similar to Iran, physicians have the lowest share in tobacco consumption. There was a statistically significant difference (*p* = 0.037) between physicians and the other two groups of European HCWs with increased use of tobacco. The highest tobacco use was detected among nurses and OHS working rotatory shifts. In contrast to Iran, their workplace had no considerable and significant effect on tobacco consumption. Similar to Iranian HCWs, according to statistics, European HCWs reported no significant change in tobacco consumption regarding gender, marital status, educational level, having chronic illness, etc.

The total numbers of HCWs who reported alcohol use were 272 (43.8%) in Iran and 348 (58.9%) in the European countries. The numbers of HCWs in Iran and the European countries who reported an increase in alcohol use due to the pandemic were 139 in Iran and 147 in the European countries. A decreased alcohol consumption was reported by 42 Iranian HCWs and 59 European ones. For 91 Iranian and 142 European HCWs, no change in alcohol use was experienced. In Iran, HCWs working in outpatient clinics reported more alcohol use in comparison with those working in clinics + departments or just departments. In the European countries, there was slightly higher alcohol consumption among HCWs working in departments. In both Iranian and European HCWs, we did not manage to find factors which considerably affect alcohol consumption at an acceptable significance level.

Volunteers with a record of psychiatric treatment were excluded in our study. Five Iranian participants and two Europeans reported suicidal history. Among Iranian HCWs, 39 active suicidal ideation was observed while this number was 12 in European HCWs. In both Iranian and European HCWs, an increase in BAI, FCV-19S, GHQ-12, and PSQI can be seen for females, but there exists no statistically significant difference. In addition, there is no significant difference in terms of quality of life (WHOQOL-BREF) in male and female HCWs in both countries. For European HCWs, the significance levels (in gender-based analysis), for BAI, FCV-19S, GHQ-12, WHOQOL-BREF, and PSQI were 0.1012, 0.0937, 0.1992, 0.2015, 0.0734, respectively. The confidence levels for Iranian HCWs for BAI, FCV-19S, GHQ-12, WHOQOL-BREF, and PSQI were 0.0986, 0.1259, 0.0873, 0.1430, and 0.0698, respectively. As can be seen in both societies, there is no significant difference between females and males suggesting that both genders in both countries follow almost the same pattern.

No significant difference was determined in terms of educational level in both countries. Iranian and European nurses experience more anxiety and distress in comparison to physicians and OHS. They also claimed lower sleep quality and a decrease in the quality of their lives. For GHQ-12, there is a significant difference between nurses and the other two groups in Iran (*p* = 0.0357) and the studied European countries (*p* = 0.0429). In Iranian nurses, a significant difference (*p* = 0.0368) exists for the BAI measure (in comparison with physicians and OHS), while there is no such difference for European nurses. While in terms of other measures, although there is an increase for nurses in both countries, there exists no statistically significant difference. In an eye-bird view, nurses experience the most stress, anxiety, fear of COVID-19, and lower quality of life and sleep in both countries. Physicians come second in Iran and the studied European countries. They report lower anxiety but almost the same distress in comparison with nurses. There is a considerable (but slight compared to nurses) decrease in the quality of sleep and life for physicians. OHS groups in both countries have reported less anxiety and stress. They experience less fear of COVID-19 in comparison with nurses but more in comparison with nurses. However, the quality of life and sleep in OHS shows a decrease like nurses. Although there are increases in almost all measures for HCWs in both countries, as was mentioned, except for GHQ-12 (for Iranian and European nurses) and BAI (for Iranian nurses), no significant difference exists between physicians, nurses, and other healthcare staff in both countries. According to the recorded measures, HCWs in Iran are under more mental pressure and psychological distress. The level of anxiety, fear, and stress is much higher in Iranian HCWs, and there is also a significant difference in the quality of sleep and life. In Iran, nurses have reported the highest psychological distress and anxiety. However, in the studied European countries, nurses and physicians experience almost the same mental pressure, anxiety, and stress.

In Iran, HCWs working in both outpatient clinics and departments have reported the highest anxiety and the lowest quality of life and sleep. However, working in just clinics or departments did not affect the level of anxiety and psychological distress. Iranian HCWs providing service at either outpatient clinics or departments claimed to have a higher quality of life and sleep. Iranian HCWs working in pandemic outpatient clinics experience the lowest anxiety, psychological distress, fear of COVID-19, sleep disturbance, and the highest quality of life. There exists a statistically significant difference between HCWs working in clinics + departments and those who work in either clinics or departments. The significance intervals were 0.0418, 0.0465, 0.0301, 0.0378, 0.0426, and 0.0213 for BAI, GHQ-12, FCV-19S, WHOQOL-BREF, and PSQI, respectively. For European HCWs, the place of service played a crucial role in the anxiety, fear, and distress they experienced. European HCWs working in outpatient clinics + departments showed higher values of BAI, GHQ-12, FCV-19s, and PSQI, respectively. Their quality of life was much lower in comparison to the other two groups suggesting there exists a statistically significant difference. The significance intervals were 0.0491, 0.0407, 0.0429, 0.0485, 0.0381, and 0.0396 for BAI, GHQ-12, FCV-19S, WHOQOL-BREF, and PSQI, respectively.

The prevalence of the measures in Iran, the above-mentioned European countries, and the full sample are described in Table 3. Measures whose *p*-values are equal or below 0.05 (*p* ≤ 0.05) are written in bold and marked by a star (^*^). In terms of FCV-19S and GHQ-12, for example, there are significant differences between Iranian and European HCWs.

**Table 3:** Prevalence of psychological distress, anxiety, the level of quality of life, and sleep problems among HCWs from Iran and the European countries.

		**Iran**			**Studied European countries**			**Full sample (Iran and the studied European countries)**				
**No**.	**Measure**	**Phs**	**Nus**	**OHS**	**Phs**	**Nus**	**OHS**	**Phs**	**Nus**	**OHS**	**All HCWs**	**Range**
1	**FCV-19S**											7–35
	Yes *N* (*n*%)	126 (70.39%)	239 (79.13%)	103 (74.10%)	105 (56.75%)	197 (70.86%)	73 (57.48%)	231 (63.46%)	436 (75.17%)	176 (66.16%)	843 (69.66%)	
	Min–Max	12–31	13–32	15–30	18–30	14–31	9–29	12–31	14–32	9–30	9–32	
	Mean ± SD	17.53 ± 5.26	22.87 ± 6.98	20.74 ± 5.83	14.51 ± 4.87	18.49 ± 5.99	19.84 ± 6.07	16.02 ± 8.63	20.68 ± 7.27	20.29 ± 7.91	18.99 ± 8.25	
	*p*	0.022*			0.035*			0.079					
2	**GHQ-12**											0–12
	Yes *N* (*n*%)	148 (82.68%)	253 (83.77%)	95 (68.34%)	112 (60.54%)	171 (61.51%)	89 (70.07%)	260 (71.42%)	424 (73.10%)	184 (69.17%)	868 (71.73%)	
	Min–Max	4–9	3–11	4–9	5–11	4–11	5–10	4–11	3–11	4–10	3–11	
	Mean ± SD	6.91 ± 2.74	8.86 ± 3.19	7.02 ± 2.55	5.97 ± 3.40	7.53 ± 2.99	6.88 ± 3.54	6.44 ± 4.87	8.19 ± 3.11	6.95 ± 3.09	7.19 ± 4.82	
	*p*	0.043*			0.049*			0.047*					
3	**BAI**											0–63
	Yes *N* (*n*%)	133 (74.30%)	268 (88.74%)	91 (65.46%)	107 (57.83%)	195 (70.14%)	81 (63.77%)	240 (65.93%)	463 (79.82%)	172 (64.66%)	875 (72.31%)	
	Min–Max	9–51	8–52	10–51	8–53	9–50	9–52	8–53	8–52	9–52	8–53	
	Mean ± SD	12.37 ± 6.29	22.51 ± 8.95	17.84 ± 5.39	11.67 ± 7.36	16.99 ± 9.58	17.86 ± 8.73	12.02 ± 8.19	19.75 ± 9.26	17.85 ± 6.73	16.54 ± 7.96	
	*p*	0.069			0.041*			0.714					
4	**WHOQOL-BREF**											26–130
	Poor *N* (*n*%)	92 (51.39%)	279 (92.38%)	112 (80.57%)	84 (45.40%)	254 (91.36%)	107 (84.25%)	176 (48.35%)	533 (91.89%)	219 (82.33%)	928 (76.69%)	
	Min–Max	39–119	36–103	33–101	45–121	40–110	37–107	39–121	36–110	33–107	33–121	
	Mean ± SD	86.42 ± 11.75	79.81 ± 15.38	82.16 ± 13.79	93.58 ± 13.09	88.71 ± 12.43	89.41 ± 14.54	90.00 ± 12.94	84.26 ± 13.69	85.78 ± 13.98	86.68 ± 13.47	
	*p*	0.061			0.052			0.046*					
5	**PSQI total**											0–21
	Poor *N* (*n*%)	147 (82.12%)	266 (80.07%)	108 (77.69%)	89 (48.10%)	253 (91.00%)	99 (77.95%)	236 (64.83%)	519 (89.48%)	207 (77.81%)	962 (79.50%)	
	Min–Max	3–16	6–18	5–15	3–15	7–18	5–16	3–16	6–18	5–16	3–18	
	Mean ± SD	4.23 ± 3.48	9.97 ± 2.56	6.07 ± 3.91	4.39 ± 4.07	6.98 ± 3.84	7.28 ± 4.17	4.31 ± 3.67	8.47 ± 3.59	6.67 ± 4.02	6.48 ± 3.79	
	*p*	0.037*			0.034*			0.041*					

Average values (Mean), standard deviation (SD), Min-Max values, and significant levels for comparison of the scale scores are represented as well.

To test the normality of the proposed measures, we conducted the normality test using Kolmogorov–Smirnov (K-S) normality test. Results are presented in Table 4. We conducted the normality analysis in Iran, European countries, and the whole samples for all HCWs. In addition, we performed the test for nurses, physicians, and other healthcare workers to clarify the distribution of the recorded scores. Scores with *p*-values below 0.01 are considered with a normal distribution. For sake of space and due to a large number of recorded scores, we reported just the number of scores in each measure whose associated *p*-values were below 0.01. As it is clear, only some of the proposed measures have the normal distribution and passed the normality tests which we expected due to the small number of samples and the limitations we had while gathering data and conducting this study.

**Table 4:** Normality test of the recorded scores using Kolmogorov–Smirnov (K-S) test.

		**Iran**			**Studied European countries**			**Full sample (Iran and the**			
								**studied European countries)**			
**No**.	**Measure**	**Phs**	**Nus**	**OHS**	**Phs**	**Nus**	**OHS**	**Phs**	**Nus**	**OHS**	**All HCWs**
1	FCV-19S	1,2,3,6,7	1,2,4,6,7	1,2,3,7	1,2,6,7	1,2,3,4,7	1,2,6,7	1,2,3,7	1,2,4,6,7	2,3,4,5	1,2,4,6,7
2	GHQ-12	1,2,6,7,9	3,8,9,11	1,2,3,8,11	1,2,3,9,11	1,2,7,9,11	1,2,3,4,9,10	6,9,11	1,3,7,8,11	2,7,9	1,3,7,8,9,11
3	BAI	3,12,15	2,3,5,7	2,7,12	6,8,15,17	2,3,19,21	9,12,21	2,3,17	2,17,21	3,16,21	2,3,6,12,17,21
4	WHOQOL-BREF	1,2,18	7,21,23	1,8,18,20	1,7,8,11	3,7,15,23	3,8,18,21,23	2,8,9,17	3,7,18,21	1,4,9,11	7,8,11,18,23
5	PSQI total	1,5,8,22	2,8,13,21	1,5,7,13,24	6,9,11,24	6,13,15,19	1,5,7,8,19	4,5,13	8,11,17,24	1,5,19	5,13,24

The numbers of the items detected with a normal distribution in each of the aforementioned measures are reported in this table.

In Iran, there is a positive correlation between age and FCV-19S (*r* = 0.129 and *p* = 0.009), between age and PSQI (*r* = 0.237 and *p* = 0.009), and between BAI and PSQI (*r* = 0.209 and *p* = 0.001). There is also a negative correlation between FCV-19s and WHOQOL-BREF (*r* = −0.0141 and *p* = 0.031), between GHQ-12 and WHOQOL-BREF (*r* = −0.361 and *p* = 0.009), and also between BAI and WHOQOL-BREF (*r* = −0.195 and *p* = 0.002). Correlation analysis for the proposed measures is represented for Iran, the studied European countries, and the full sample in Tables 5–**7**, respectively. Correlations with a *p*-value equal to or below 0.05 (*p* ≤ 0.05) are written in bold and marked by a star (^*^).

**Table 5:** Correlation analysis between our suggested measures (Iran).

**Measures**		**Age**	**FCV-19S**	**GHQ-12**	**BAI**	**WHOQOL-BREF**	**PSQI total**
Age	*r*	1	0.129*	0.408	−0.125	0.093	0.237*
	*p*		0.009*	0.136	0.631	0.768	0.009*
FCV-19S	*r*	0.129*	1	0.028	0.107	–0.141*	0.326
	*p*	0.009*		0.061	0.102	0.031*	0.063
GHQ-12	*r*	0.408	0.028	1	0.116	–0.361*	0.133
	*p*	0.136	0.061		0.079	0.009*	0.058
BAI	*r*	−0.125	0.107	0.116	1	–0.195*	0.209*
	*p*	0.631	0.102	0.079		0.002*	0.001*
WHOQOL-BREF	*r*	0.093	–0.141*	–0.361*	–0.195*	1	−0.278
	*p*	0.768	0.031*	0.009*	0.002*		0.087
PSQI total	*r*	0.237*	0.326	0.133	0.209*	−0.278	1
	*p*	0.009*	0.063	0.058	0.001*	0.087	

Among the studied European countries, as can be seen in Table 6, there is a positive correlation between age and GHQ-12 (*r* = 0.287 and *p* = 0.041), between age and PSQI (*r* = 0.236 and *r* = 0.027), between GHQ-12 and PSQI (*r* = 0.086 and *p* = 0.009), and between BAI and PSQI (*r* = 0.291 and *r* = 0.007). On the other hand, there is a negative correlation between age and BAI (*r* = −0.197 and *p* = 0.036), between FCV-19S and WHOQOL-BREF (*r* = −0.021 and *p* = 0.030), and between BAI and WHOQOL-BREF (*r* = −0.175 and *p* = 0.012).

**Table 6:** Correlation analysis between our suggested measures (the studied European countries).

**Measures**		**Age**	**FCV-19S**	**GHQ-12**	**BAI**	**WHOQOL-BREF**	**PSQI total**
Age	*r*	1	0.204	0.287*	−0.197*	0.093	0.236*
	*p*		0.037	0.041*	0.036*	0.428	0.027*
FCV-19S	*r*	0.204	1	0.047	0.105	−0.021*	0.329
	*p*	0.037		0.021	0.207	0.030*	0.051
GHQ-12	*r*	0.287*	0.047	1	0.081	**–**0.139	0.086*
	*p*	0.041*	0.021		0.019	0.064	0.009*
BAI	*r*	−0.197*	0.105	0.081	1	−0.175*	0.291*
	*p*	0.036*	0.207	0.019		0.012*	0.007*
WHOQOL-BREF	*r*	0.093	−0.021*	**–**0.139	−0.175*	1	**–**0.196
	*p*	0.428	0.030*	0.064	0.012*		0.139
PSQI total	*r*	0.236	0.329	0.086	0.291*	−0.196	1
	*p*	0.027	0.051	0.009	0.007*	0.139	

According to Table 7, it was determined that there was a positive correlation between age and FCV-19S (*r* = 0.236 and *p* = 0.012), also between age and PSQI (*r* = 0.193 and *p* = 0.016), between FCV-19S and PSQI (*r* = 0.182 and *p* = 0.001), between BAI and GHQ-12 (*r* = 0.097 and *p* = 0.021) and between BAI and PSQI (*r* = 0.302 and *r* = 0.002). There was also a negative correlation between GHQ-12 and WHOQOL-BREF (*r* = −0.129 and *p* = 0.006) and between BAI and WHOQOL-BREF (*r* = −0.286 and *p* = 0.001).

**Table 7:** Correlation analysis between our suggested measures (full sample).

**Measures**		**Age**	**FCV-19S**	**GHQ-12**	**BAI**	**WHOQOL-BREF**	**PSQI total**
Age	*r*	1	0.236*	0.101	−0.099	0.087	0.193*
	*p*		0.012*	0.071	0.941	0.592	0.016*
FCV-19S	*r*	0.236*	1	0.032	0.086	−0.019	0.182*
	*p*	0.012*		0.073	0.141	0.034	0.001*
GHQ-12	*r*	0.101	0.032	1	0.097*	–0.129*	0.095
	*p*	0.071	0.073		0.021*	0.006*	0.012
BAI	*r*	−0.099	0.086	0.097*	1	–0.286*	0.302*
	*p*	0.941	0.141	0.021*		0.001*	0.002*
WHOQOL-BREF	*r*	0.087	−0.019	–0.129*	–0.286*	1	−0.104
	*p*	0.592	0.034	0.006*	0.001*		0.027
PSQI total	*r*	0.193*	0.182*	0.095	0.302*	−0.104	1
	*p*	0.016*	0.001*	0.012	0.002*	0.027	

## Discussion

The present study explores the most important measures regarding mental pressure, psychological distress, anxiety, quality of life, and sleep with respect to sociodemographic data recorded from HCWs in two target populations of HCWs including Iran and the studied European countries. In this study, we tried our best to fairly compare these two populations through the aforementioned measures. The present study identified almost 79 and 70% fear of COVID-19 among Iranian and European nurses, respectively. About 80 and 90% of Iranian and European nurses claim that they suffer from poor quality of sleep. Considering the results mentioned before, it should be noted that Iranian HCWs experience a worse situation considering all measures such as BAI, GHQ-12, FCV-19S, etc. Our findings are consistent with previously published studies in the same target populations such as Motahedi et al. (79). For instance, in Iran, female nurses with a record of COVID-19 infection showed higher anxiety levels. In another study conducted in Turkey (1) with 140 participants, 70% of HCWs including nurses, physicians, and other healthcare staff reported different levels of anxiety from mild to severe which is close to our results obtained from Iran. Our gender-based comparison showed that in general female HCWs showed higher anxiety and fear of COVID-19 in Iran and those European countries alike. It is again in agreement with several studies such as (1, 2, 8, 10). In addition, in correlation analyses reported in Tables 4–6, the results are inconsistent with (1). For example, in that study and the present paper, a significant correlation exists between age and FCV-19S. Moreover, BAI and PSQI measures showed a positive correlation in Iranian, European, and Turkish HCWs. In addition to neurophysiological and neuropsychological studies, this association has also been approved that the level of anxiety is associated with the quality of life and sleep (1, 2, 8).

There was a considerable significant correlation between FCV-19S, BAI, GHQ-12, and PSQI. This shows that quality of sleep has been more affected in the pandemic in comparison to the quality of life. Most Iranian and European HCWs have claimed to suffer from poor sleep quality. Working hard for long hours, being exposed to the disease, witnessing death in each work shift, etc. were the main reasons for HCWs to be stress-out, feel anxiety, and suffer from poor sleep quality. During pandemics, governments should take serious actions to lower the workload to help HCWs as individuals fighting the disease on the front line. Poor sleep quality results in inaccuracy, making mistakes, and lower performance.

Iranian HCWs used to experience tough situations and have not been satisfied with their jobs. They report suffering from too much workload, working for long hours, no appropriate salaries, low socioeconomic status in society, no job security, etc. Recently, several studies and news have reported suicides among Iranian HCWs. As there was almost no hope to receive surefire treatment for the disease and also vaccines to get immune, Iranian HCWs were under more psychological distress. However, European HCWs were more positive about receiving vaccines at the time of recording data.

It should be noted that we gathered data during a period when HCWs are vulnerable to fatigue due to social restrictions. This can significantly affect the recorded measures such as the level of anxiety and the quality of life and sleep. As it is mentioned in Manchia et al. (80), fear of COVID-19 and psychological distress (and subscales of fatigue, anxiety, and depression) were reported to be higher among healthcare than non-healthcare workers. In a longitudinal study exploring Japanese healthcare personnel, indices of fatigue, anxiety, and depression showed an increase among health care compared to non-healthcare workers during the COVID-19 outbreak. While some studies like (81) reported a statistically significant relationship between stress, anxiety, fear of COVID-19, and fatigue, other studies such as Kachadourian et al. (82) found that there is no significant correlation between stress and anxiety and fatigue among HCWs. The authors in Kachadourian et al. (82) analyzed post-trauma disorder, major depressive disorder, and generalized anxiety disorder and the association between these psychological disorders and burnout and the occupational difficulties they have faced in 787 HCWs during the pandemic. It is reported that having tiredness/low energy, feeling tired, negative expectations, loss of interest little energy, and feeling easily annoyed or irritable are significantly associated with burnout. Due to social restrictions, it was highly probable that our studied HCWs and their subjective reports for anxiety and stress levels were correlated with fatigue. We are going to analyze the effects of burnout and fatigue on quality of life and sleep in our future study.

As was discussed, we decided to employ the most effective measures suggested by several previous studies like (1–7) to precisely quantify the level of stress and anxiety in HCWs. Previous studies have reported that these measures can appropriately describe psychological distress and anxiety. We also decided to compare the two countries to conduct a comprehensive study.

Despite all the limitations we had in this project, we managed to conduct a comprehensive study with a rich dataset compared to previous studies. For example, the authors in Korkmaz et al. (1) performed a statistical analysis to explore the relationship between anxiety, stress, quality of sleep, and life with problem-solving skills. However, only 140 HCWs participated in this study which might increase the possibility of biased results. In a systematic review and meta-analysis (79), the prevalence of anxiety, depression, acute stress, post-traumatic stress, and sleep disorders was estimated by considering 70 studies including 101,017 HCWs. The estimated prevalence was 30% of anxiety (95%CI, 24.2–37.05); 31% of depression (95%CI, 25.7–36.8); 56% of acute stress (95%CI-−30.6–80.5); 20% of post-traumatic stress (95%CI, 9.9–33.0); and 44% of sleep disorders (95%CI, 24.6–64.5). It was also reported that three factors including the proportion of females, nurses, and location are the sources of heterogeneity. In another meta-analysis (80), mental disorders among nurses were explored previously published studies the overall prevalence of stress was 43% (95% CI, 37–49), and the prevalence of anxiety was 37% (95% CI, 32–41), and the prevalence of depression the prevalence of 35% (95% CI 31–39) was reported in 40, 73, and 62 studies, respectively. In our study, the prevalence of fear of COVID-19, psychological distress, anxiety, poor quality of life, and sleep was about 68–78% according to Table 3.

Considering the results of the present study, it is worth mentioning that Iranian nurses experienced more stressful situations and report more anxiety and stress and lower quality of life and sleep. Due to their insufficient salaries and also economic inflation, Iranian nurses and OHS tend to work in two or even three healthcare centers which play a crucial role in tolerance and the level of anxiety. They are highly exposed to patients and the disease. Some Iranian nurses and OHS have reported that they have not met their family members for months. Iranian physicians reported less anxiety and distress as some of them have quit their jobs in order not to be exposed to the disease.

In pandemics, almost the whole society is affected, and HCWs who work on the front line are more prone to be infected by patients and thus are more stressed out. Intensive workload, several responsibilities, risk of exposure, etc. sharply increase in such crises. Serious actions should be taken by HCWs to protect themselves, and their family members which again leads to more stress and anxiety. In addition, they need to work hard to inform society about the disease and corresponding preventive actions. Moreover, HCWs need to be focused to use medical resources appropriately which, during pandemics and considering a load of patients, might result in more mental pressure. HCWs need to work harder during extended working hours when they witness more death. Several studies such as (1, 2, 5, 7, 34), as well as the present study, have reported that HCWs responsible for the diagnosis, treatment, and care of COVID-19 patients have exhibited sleep disturbance, distress symptoms, lower quality of life, and higher levels of depression. This implies that, in pandemics, it is vital to pay more attention to HCWs' mental health as well as their physical health to manage to be successful in health management. This aspect has been ignored by several governments and organizations. Although they suppose medical resources play the most important role in pandemics, however, human resources are key to success in such crises.

In addition to psychological symptoms and disorders caused by COVID-19, several studies such as (83–86) worked on different aspects which are quite important as well and should not be taken for granted. The authors in (83, 84), for example, assessed the potential consequences of the outbreak on gender equality in Europe. In Gómez-Salgado et al. (85), psychological distress during the COVID-19 pandemic was analyzed through a large size of the sample (4,180 individuals) in Spain. The studied parameters were sociodemographic variables, health conditions, psychological adjustment, physical symptoms, and COVID-19 contact records. Similar to our procedure, General Health Questionnaire (GHQ-12) was also employed in this study suggesting the popularity of this questionnaire to measure general health parameters. In Domínguez-Salas et al. (86), psychological distress caused by the COVID-19 pandemic was studied in a sample of the Spanish population (4,615 individuals). This distress can identify several important factors such as the predictive nature of the information received, the preventive measures taken, level of concern, beliefs, and knowledge about the infection. These studies (85, 86) suggest that more investigation and deeper analysis are required to comprehensively explore this pandemic, its predominant factors, and its side effects. It is highly recommended that future studies focus on this issue.

## Limitations of the study

The present study suffers from some limitations. In this study, we focused on the level of self-reported symptoms and just employed online questionnaires and also self-report data which can be questioned. Due to the limitations imposed by the recent pandemic, we did not manage to utilize diagnostic tools or methods. Evaluation by an external and expert observer will result in more accurate and precise results. HCWs filled out the questionnaires online and under no supervision and submitted measures might be doubted since HCWs might have filled out forms in different mental states or when they were stressed out or tired of work. These factors affect HCWs, and they might take sides and fill the forms with mental bias.

In addition, the fact of having a convenience sample should be noted as a limitation in our study. We used several applications to spread the questionnaires, and sometimes, it was a bit difficult to answer our participants' concerns appropriately. Gathering data in such studies like ours is challenging especially in terms of data collection from several districts all over the world. Moreover, there also existed some limitations regarding our methodology such as the short period of time for data collection and also the possibility of getting infected by COVID-19 during the data collection phase. Our proposed method can appropriately describe and compare HCWs in the two target populations; however, more analysis is required to fully explore the causes of low life and sleep quality and the relationship and correlation between the denominators and these important qualities of life and sleep. It is worth mentioning that another significant limitation was regarding the representativeness of respondents who were sometimes difficult to reach out to. Finding eligible participants from our target districts was one of our challenges and took a considerable amount of time. Although we have tried our best to collect as larger datasets as possible but larger datasets will open up new horizons and will result in new findings in future studies.

## Data availability statement

The raw data supporting the conclusions of this article will be made available by the authors, without undue reservation.

## Ethics statement

The studies involving human participants were reviewed and approved by Research Ethics Committee of Islamic Azad University—Science and Research Branch. The patients/participants provided their written informed consent to participate in this study.

## Author contributions

MoZ proposed the first idea and managed the whole project. MoZ, PT, SK, MSK, SJ, and AS collected the data and performed data collection and analysis. MoZ, PT, MSK, and AS carried out statistical analysis and prepared the repots. MoZ, KM, MaZ, SJ, and NJD conducted data analysis and reviewed the results. MoZ and PT wrote the manuscript and edited it. MaZ, KM, and NJD supervised the project. All authors reviewed the results and the manuscript.

## Conflict of interest

The authors declare that the research was conducted in the absence of any commercial or financial relationships that could be construed as a potential conflict of interest.

## Publisher's note

All claims expressed in this article are solely those of the authors and do not necessarily represent those of their affiliated organizations, or those of the publisher, the editors and the reviewers. Any product that may be evaluated in this article, or claim that may be made by its manufacturer, is not guaranteed or endorsed by the publisher.
